# Major Complex Abdominal Wall Repair in Contaminated Fields with Use of a Non-cross-linked Biologic Mesh: A Dual-Institutional Experience

**DOI:** 10.1007/s00268-017-3962-2

**Published:** 2017-03-06

**Authors:** J. J. Atema, E. J. Furnée, Y. Maeda, J. Warusavitarne, P. J. Tanis, W. A. Bemelman, C. J. Vaizey, M. A. Boermeester

**Affiliations:** 1 Department of Surgery, Academic Medical Centre, 1105 AZ Amsterdam, The Netherlands; 2 Department of Surgery, St Mark’s Hospital, London, UK

## Abstract

**Background:**

Data on the use of biologic mesh in abdominal wall repair in complex cases remain sparse. Aim of this study was to evaluate a non-cross-linked porcine acellular dermal matrix for repair of complex contaminated abdominal wall defects.

**Methods:**

Retrospective observational cohort study of consecutive patients undergoing abdominal wall repair with use of Strattice™ Reconstructive Tissue Matrix (LifeCell Corporation, Oxford, UK) between January 2011 and February 2015 at two National Intestinal Failure Units.

**Results:**

Eighty patients were identified. Indications for abdominal wall repair included enterocutaneous fistula takedown (*n* = 50), infected synthetic mesh removal (*n* = 9), restoration of continuity or creation of a stoma with concomitant ventral hernia repair (*n* = 12), and others (*n* = 9). The median defect area was 143.0 cm^2^ (interquartile range or IQR 70.0–256.0 cm^2^). All had a grade III or IV hernia. Component separation technique (CST) was performed in 54 patients (68%). Complete fascial closure was not possible despite CST and biologic mesh-assisted traction (bridged repair) in 20 patients (25%). In-hospital mortality was 1%. Thirty-six patients (45%) developed a wound infection. None required mesh removal. Of 76 patients with a median clinical follow-up of 7 months (IQR 4–15) available for analysis, 10 patients (13%) developed a hernia recurrence, of whom 3 had undergone bridged repairs. Seven patients developed a postoperative (recurrent) fistula (9%).

**Conclusion:**

Repair of challenging and contaminated abdominal wall defects can be done effectively with non-cross-linked biologic mesh and component separation technique without the need for mesh removal despite wound infections.

## Introduction

Synthetic mesh repair is generally accepted as the preferred treatment strategy for clean abdominal wall defects. However, the use of synthetic material is frequently perceived as contraindicated for more complex cases, especially in the presence of contamination. The introduction of biologic prosthetics has provided new meshes that have the potential to resist infection [1, 2]. Numerous biologic prostheses have been developed using human or animal source material and different processing techniques such as collagen cross-linking. There have been multiple studies reporting their use in abdominal wall repair [3–5]. There is no general agreement on the indications for the use and cost-effectiveness of these meshes [6]. Most authors advocate the use of biologic mesh in “difficult” situations. However, no consensus exists on the definition of a difficult or complex hernia [7]. The utility of biologic meshes in contaminated fields is difficult to determine from the existing literature as most studies included simple or clean hernias [8]. There remains some concern about the use of a cross-linked biologic mesh in contaminated areas [9, 10]. As not all biologic meshes behave in the same way, each needs individual evaluation [11, 12].

The primary endpoint of this study was to evaluate the results of abdominal wall repair using a single biologic mesh (non-cross-linked porcine dermal matrix) in a homogeneous series of patients with major complex and contaminated abdominal wall defects.

## Methods

All consecutive patients undergoing elective repair of a ventral abdominal wall defect with use of a non-cross-linked porcine biologic mesh (STRATTICE™ Reconstructive Tissue Matrix, LifeCell Corporation, Branchburg, NJ, USA) between January 2011 and February 2015 at the Academic Medical Centre (Amsterdam, The Netherlands) and St. Marks Hospital (London, UK), two established European intestinal failure centres, were included in this retrospective observational cohort study. In both centres, a biologic mesh was used only in patients with contaminated abdominal wall defects. The decision to use a biologic mesh was made intraoperatively and was left to the discretion of the surgeon.

### Data collection, variables and definitions

Eligible patients were identified from an administrative surgical registry in both centres and in the Academic Medical Centre Amsterdam from a prospective database of patients with intestinal failure and abdominal wall defects. Data were gathered retrospectively from medical records and included patient characteristics, abdominal wall defect characteristics, surgical details, postoperative morbidity and outcome. The abdominal wall defects were graded according to the Ventral Hernia Working Group (VHWG) grading system [13]. Additionally, hernias were assigned to one of three severity classes (minor, moderate and major complex) described by an expert consensus group in 2014 [7]. Hernia size was calculated based on preoperative imaging. Postoperative wound infections were divided into minor and major. A minor wound infection was defined as any infection of the surgical wound that could be managed conservatively, with antibiotics or by opening at the bedside, whereas a major wound infection was defined as requiring percutaneous or surgical intervention. Postoperative morbidity was graded according to the Clavien–Dindo classification, with grade III or higher regarded as major complications [14].

Recurrence of hernia and fistula was assessed clinically and/or radiologically. A ventral hernia recurrence was diagnosed by physical examination and/or imaging with ultrasound or computed tomography (CT). When primary fascial closure was achieved, an abnormal contour without a fascial defect was defined as bulging or laxity. An enteric fistula recurrence was defined as any defect of the abdominal wall with apparent enteric output, if necessary confirmed by imaging (CT or contrast radiography).

### Surgical strategy

**Fig. 1:**
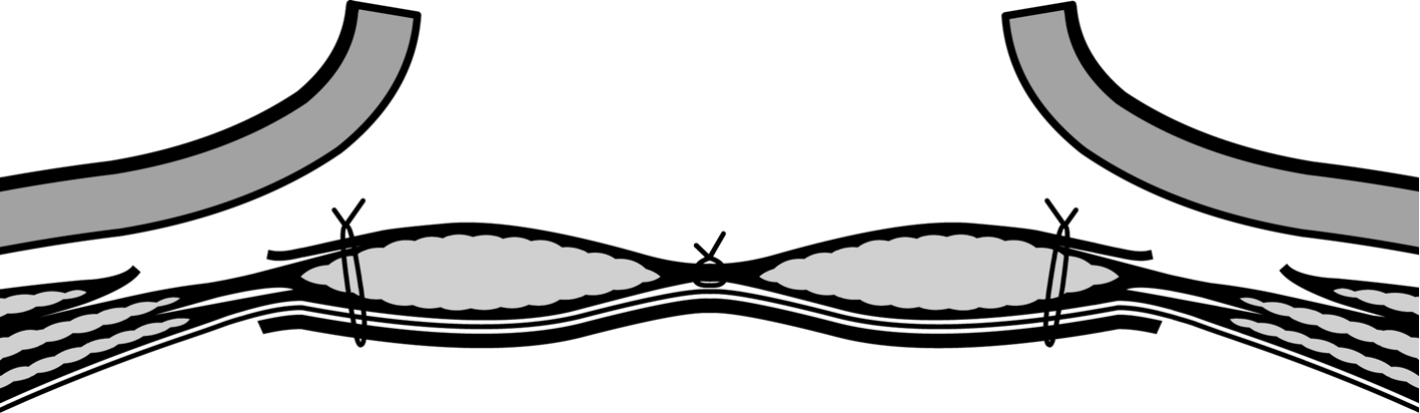
Schematic illustration of a reinforced repair with an intraperitoneally placed biologic mesh and component separation technique to enable primary fascial closure

The prerequisites for reconstructive surgery were resolution of abdominal sepsis and optimised nutritional status with use of enteral or parenteral nutritional support. Generally, surgery was delayed for a period of 6 months after the last abdominal intervention. Surgical procedures included complete adhesiolysis with bowel and other viscera being dissected free from the abdominal wall. Surgical treatment of enteric fistula involved resection with the construction of an anastomosis. Perioperative bowel preparation was not routinely administered. Enemas were given when the operating surgeon felt it was needed for left-sided colorectal resections. In cases with infected synthetic mesh, all previously inserted synthetic material was removed whenever possible. In all patients, every attempt was made to achieve primary fascial closure. If primary closure was not possible without undue tension, a component separation technique (CST) was performed, mostly by vertical transection of the aponeurosis of the external oblique muscle and separation of the external oblique muscle from the internal oblique [15]. Biologic meshes were preferably positioned intraperitoneally (intraperitoneal onlay mesh or IPOM) and were sutured under tension to distribute forces evenly and to facilitate primary fascial closure (Fig. 1). Alternatively, meshes were placed as onlay, inlay or in a retro-rectus position depending on the quality of fascia and available space for mesh placement. During the more recent reconstructions, a (partly) absorbable synthetic mesh (Vypro or Phasix) was considered for additional onlay reinforcement in case of a bridging biomesh (i.e. when midline closure failed despite a CST). Intravenous antibiotic prophylaxis covering aerobic and anaerobic bacteria was administered to every patient. Intra-abdominal drains were not routinely places. If a CST was performed, subcutaneous suction drains were placed, which were removed when production was less than approximately 30 cc per 24 h. Postoperatively, patients were instructed to use a hernia belt for the first 3 months during mobilisation. Postoperative intra-abdominal infectious complications, including anastomotic leakage, were treated non-surgically whenever possible with percutaneous drainage and antibiotics. During reoperations, the biologic mesh was left in place, was opened in midline and closed by a running suture.

### Statistical analysis

Normally distributed continuous data were expressed as mean (standard deviation or SD) and non-normally distributed data as median (range or interquartile range [IQR]). Testing for normality was done with the Shapiro–Wilk test. Data handling and analyses were done with SPSS^®^ software version 20.0 (IBM, Armonk, New York, USA). Outcomes were reported for overall, reinforced and bridged repairs. Continuous data were compared with the independent *t* test or the Mann–Whitney U test; categorical data were compared with Chi-square test or the Fisher’s exact test.

### Ethical consideration

The manuscript was written in accordance with the Strengthening the Reporting of Observational Studies in Epidemiology (STROBE) statement [16]. The Medical Ethics Board of the Amsterdam Medical Centre approved the study protocol and waived the need for informed consent. Approval for use of retrospective clinical data and publication was granted by the Research and Development Department at London North West Healthcare NHS Trust.

## Results

### Patients

A total of 80 patients met the inclusion criteria and were included. Patient and abdominal wall defect characteristics are summarised in Table 1. Median age of the patients was 63 years (IQR 48–69 years), and 47 (59%) were male. Patients had previously undergone a median number of 4 abdominal operations (range 1–25), and 50% of the included patients (40 of 80) had a history of an open abdomen. Indications for abdominal wall reconstruction included enterocutaneous or enteroatmospheric fistula takedown (*n* = 50), infected synthetic mesh removal (*n* = 9), restoration of continuity or creation of a stoma with concomitant ventral hernia repair (*n* = 12) and others (*n* = 9). All patients had a VHWG Grade III or IV abdominal wall defect [13]. Eighty-two per cent (71 of 80) of the patients had a hernia that could be classified as a major complex abdominal wall defect [7]. Patients in whom a fascial gap needed to be bridged more frequently had a stoma compared to patients with primary fascial closure (*p* = 0.020). Furthermore, all patients with a grade IV hernia had reinforced (non-bridging) repairs (*p* = 0.016).

**Table 1:** Patient and abdominal wall defect characteristics

	All (*n* = 80)		Reinforced repairs (*n* = 60)		Bridged repairs (*n* = 20)		*p* value
Age in years, median (IQR)	63	(48–69)	63	(51–70)	52	(41–68)	0.083
Male gender	47	(59%)	37	(62%)	10	(50%)	0.359
ASA classification, median (range)	2	(1–3)	2	(1–3)	2	(1–3)	1.000
Body mass index in kg/m^2^, mean (±SD)	27.8	(±5.9)	26.6	(±5.8)	28.0	(±6.2)	0.796
Serum albumin in g/L, mean (±SD)^‡^	35	(±9)	35	(±9)	34	(±11)	0.788
Smoking status	18	(23%)	14	(23%)	4	(20%)	0.690
Diabetes	20	(25%)	15	(25%)	5	(25%)	1.000
Cardiac comorbidity	21	(26%)	17	(28%)	4	(20%)	0.566
Pulmonary comorbidity	17	(21%)	14	(23%)	3	(15%)	0.540
Inflammatory bowel disease	13	(16%)	9	(15%)	4	(20%)	0.727
Preoperative need for parenteral nutrition	30	(38%)	23	(38%)	7	(35%)	1.000
Number of previous abdominal surgeries, median (range)	4	(1–25)	4	(1–15)	5	(1–25)	0.951
History of open abdomen	40	(50%)	29	(48%)	11	(55%)	0.169
Defect area in cm^2^, median (IQR)	143.0	(70.0–256.0)	146.5	(69.0–224.0)	88	(63.0–297)	0.778
Defect width in cm, median (IQR)	11.0	(6.0–14.8)	10.0	(6.0–14.0)	8.0	(5.8–15.3)	0.799
Stoma present	37	(46%)	23	(38%)	14	(70%)	*0.020*
Enterocutaneous or enteroatmospheric fistula	50	(63%)	40	(67%)	10	(50%)	0.182
Ventral hernia working group grade [13]							*0.016*
I/II	0	(0%)	0	(0%)	0	(0%)	
III	66	(83%)	46	(77%)	20	(100%)	
IV	14	(18%)	14	(23%)	0	(0%)	
Hernia complexity class [11]							0.684
Minor complex	0	(0%)	0	(0.0%)	0	(0%)	
Moderate complex	9	(11%)	9	(15%)	7	(35%)	
Major complex	71	(89%)	51	(85%)	13	(65%)	

Values in italic indicates statistical significance (*p* < 0.05)

*IQR* interquartile range, *SD* standard deviation

^‡^ Serum albumin level was available for 51 patients (39 reinforced and 12 bridged repairs)

### Surgery

Details of the 80 surgical procedures are given in Table 2. In 60 patients (75%), concomitant gastrointestinal surgery was performed with the construction of one or more intestinal anastomoses (median 1, range 1–4 anastomoses). Synthetic mesh was removed in 27 (34%) patients of whom 9 had infected mesh removed. Component separation was performed to obtain primary closure or to minimise the remaining fascial defect in 55 patients (69%), bilaterally in 46 patients. The biologic mesh was placed in an intraperitoneal position in the majority of patients (67/80; 84%) (Fig. 1). In 5 patients (6%), a partly absorbable synthetic lightweight multifilament mesh (Vypro™, Ethicon, Norderstedt, Germany) was used to enhance the repair, either as onlay (3) or in a retro-rectus position (2). Primary fascial closure was achieved in 59 patients (74%), while in 20 patients (25%) the biologic mesh was used to bridge a fascial gap. Whether or not fascial closure was achieved was unclear in one patient. Soft tissue closure was achieved in all patients, with local skin and subcutaneous tissue advancement performed by a plastic surgeon in 8 patients (10%), and a pedicled regional flap (m. tensor fasciae latae) in 2 patients (3%).

**Table 2:** Operative details and postoperative morbidity

	All (*n* = 80)		Reinforced repairs (*n* = 60)		Bridged repairs (*n* = 20)		*p* value
Operation time in minutes, median (IQR)	370	(256–449)	355	(241–435)	408	(351–551)	*0.028*
Anastomosis constructed	60	(75%)	43	(72%)	17	(85%)	0.372
Synthetic mesh removed	27	(34%)	24	(40%)	3	(15%)	0.065
Component separation technique performed	55	(69%)	40	(67%)	15	(75%)	0.585
Mesh positon							0.015
Unclear	2	(3%)	2	(3%)	0	(0%)	
Onlay	4	(5%)	4	(7%)	0	(0%)	
Inlay	3	(4%)	0	(0%)	3	(15%)	
Retro-rectus	4	(5%)	4	(7%)	0	(0%)	
IPOM	67	(84%)	50	(83%)	17	(85%)	
Fascial closure							0.000
Unclear	1	(1%)	1	(2%)	0	(0%)	
Primary fascial closure (mesh reinforcement)	59	(74%)	59	(98%)	0	(0%)	
Bridging mesh	20	(25%)	0	(0%)	20	(100%)	
Soft tissue closure	80	(100%)	60	(100%)	20	(100%)	1.000
Any postoperative complication	59	(74%)	43	(72%)	16	(80%)	0.566
Minor wound infection	26	(33%)	20	(33%)	6	(30%)	1.000
Major wound infection	10	(13%)	7	(12%)	3	(15%)	0.705
Pneumonia	23	(29%)	17	(28%)	6	(30%)	1.000
Anastomotic leakage	6	(10%)^‡^	3	(7%)^‡^	3	(18%)^‡^	0.338^‡^
Intra-abdominal abscess	12	(15%)	9	(15%)	3	(15%)	1.000
Postoperative enterocutaneous fistula	7	(9%)	3	(5%)	4	(20%)	0.108
Reoperation within index admission	3	(4%)	3	(5%)	0	(0%)	0.567
Unplanned IC admittance	16	(20%)	9	(15%)	7	(35%)	0.102
Complication of grade III or IV according to Clavien–Dindo [22]	29	(36%)	17	(28%)	12	(60%)	*0.016*
Length of postoperative hospital stay in days, median (range)	15	(4–121)	15	(4–112)	20	(7–121)	0.210
Readmission rate within 30 days	22	(28%)	16	(27%)	6	(30%)	0.772
In-hospital mortality	1	(1%)	1	(2%)	0	(0%)	1.000

Values in italic indicates statistical significance (*p* < 0.05)

*IQR* interquartile range, *IPOM* intraperitoneal onlay mesh

^‡^ Percentage of all patients with a constructed intestinal anastomosis

### Complications

Fifty-nine patients (74%) developed one or more postoperative complications. Twenty-six patients (33%) developed a minor wound infection and ten patients (13%) a major wound infection. Of the 60 patients in whom one or more intestinal anastomoses were constructed, six (10%) developed anastomotic leakage. An intra-abdominal abscess was diagnosed in 12 patients (15%). The readmission rate was 28%, with most readmissions being related to wound infections. Three patients (4%) underwent a reoperation during the index admission. Two reoperations were performed because of a wound infection, and one patient underwent reoperation with an additional biologic mesh after developing a recurrent enterocutaneous fistula due to an anastomotic leakage within 2 weeks after the index fistula resection. No biologic mesh needed removal in any patient. One patient died 13 days postoperatively due to abdominal sepsis and multi-organ failure as a result of an anastomotic leakage, resulting in an in-hospital mortality rate of 1%. Compared to reinforced repairs, bridged repairs were associated with a longer duration of surgery (*p* = 0.028) and a higher rate of postoperative grade III or IV complications according to Clavien–Dindo (*p* = 0.016).

### Clinical follow-up

Three patients (4%) were lost to follow-up after discharge and one patient died during the index admission, leaving a total of 76 patients available for further analyses. Median duration of clinical follow-up for these 76 patients was 7 months (IQR 4–15). Ten patients (13%) developed a recurrent ventral hernia. Six of these patients had primary fascial closure at initial abdominal wall repair, whereas three patients had a remaining fascial gap despite CST. In the last patient with a clinical hernia recurrence, it was not clear whether or not fascial closure was achieved. Five of the 10 patients remained asymptomatic; therefore, their recurrent hernias were not repaired. The remaining five patients with a recurrent hernia underwent surgical repair with use of a biologic mesh (3), a synthetic mesh (1) or primary repair (1). Another two (3%) patients with initial primary fascial closure had bulging of the abdominal wall at physical examination during clinical follow-up without signs of a fascial defect.

Of the seven patients (9%) who developed an enterocutaneous fistula postoperatively, six had initially undergone fistula surgery. Initial fascial closure had been achieved in three patients, whereas a fascial gap had been bridged in four. Three patients were successfully managed conservatively, three patients underwent surgical fistula takedown with concomitant abdominal wall repair, and one patient is booked for surgery in the near future.

## Discussion

Abdominal wall repair with concomitant enteric fistula takedown or removal of an infected synthetic mesh is known to be associated with significant complications. This series demonstrated that repair of such complex contaminated defects with non-cross-linked biologic mesh can be done safely and effectively. Removal of the mesh was never necessary, and the rate of hernia recurrence was 13% during a median clinical follow-up of 7 months.

The rate of wound infections in our complex series of patients was 45%, which falls within range of previously described rates following repair of defects in the presence of contamination (35.0–47.7%) [17–21]. Despite this high rate of wound infections, removal of the mesh was not necessary in any patient. Synthetic mesh infection is a feared complication, although favourable outcome of lightweight synthetic mesh repair in the presence of contamination has been reported [22]. In this series by Carbonell et al., the wound infection rate was acceptable with only four meshes (4%) being removed and a hernia recurrence rate of 7% with a mean follow-up of 10.8 months. The comparability of this study to this present series is questionable, as only few patients presented with concomitant fistula or had an infected synthetic mesh.

This series represents a challenging patient population. Comparison of our results to other series is hindered by the fact most previous reports have included patients with varying levels of wound contamination or failed to adequately describe the complexity of the hernias [18, 23–26]. A prospective multi-centre study on the use of a non-cross-linked porcine dermal matrix in patients with lower complexity reported a hernia recurrence rate of 28% after 2 years [19]. A case series evaluating the results of enterocutaneous fistula takedown and simultaneous abdominal wall reconstruction with use of a biologic mesh, the vast majority non-cross-linked porcine derived, reported a hernia recurrence of 32% after a mean follow-up of 20 months [27]. In another study describing reconstructive surgery for intestinal fistula in an open abdomen, a small portion of patients underwent cross-linked porcine mesh repair [21]. Hernia and fistula recurrence rates were both as high as 41.7%. Suture repair showed favourable outcomes, although selection bias was clearly present. Compared to existing literature, our results show good outcome in this challenging group of complex patients. It has been demonstrated that recurrences may develop several years after reconstruction, so our recurrence rate is expected to rise [17].

In this study, a primary fascial closure rate of 80% and a 100% abdominal cavity closure rate were accomplished. Given that half of all patients had an open abdomen prior to definitive surgery, this was not achievable without the use of biologic mesh achieving traction to the midline to close extremely large fascial defects with the combination of CST. Anastomotic leakage is anticipated to be lower in a closed abdomen; this is illustrated by an anastomotic leakage rate of only 10% in the present complex reconstruction series.

Bridged repairs are known to offer inferior results compared to mesh-reinforced repairs with midline closure [23]. In our series, 20 patients (25%) had a bridged repair because of a remaining fascial gap despite component separation or because effective component separation was not possible. Of these, 15% had a recurrent hernia at clinical follow-up compared to 10% of the patients with a reinforced repair. This relatively low recurrence rate is partly explained by the limited follow-up. This rate is comparable to a study on 37 bridged repairs with a biologic mesh, reporting a recurrence rate of 19% with a mean follow-up of 13 months [28]. However, much higher recurrence rates have also been reported ranging from 56 to 89%, which calls into question whether bridging a fascial gap with a biologic mesh offers advantages over the use of an absorbable mesh; results are likely to differ among various biologic meshes manufactured with different processing techniques [23, 29, 30].

Several limitations of the present study need to be addressed. As it was retrospective, there were some missing data and possible attrition bias. Another drawback of this manuscript is the median clinical follow-up of less than 1 year, and recurrence rate is likely to increase with longer follow-up. However, the crucial outcome of these complex repairs lies in the initial postoperative period of this one-stage repair when anastomoses and abdominal wall wounds need to heal without long-lasting complications such as anastomotic leakage and wound dehiscence. Not all patients underwent routine diagnostic imaging to detect hernia recurrences. Imaging was only performed when a recurrent hernia was suspected clinically. Hernia recurrence is likely to be rated more often by imaging, but less clinically relevant and in part less accurate because in case of biologic meshes the interpretation of the newly formatted fibrotic tissue lining at the site of the biomesh position is more difficult to interpret. As this is a series of the use of a single biologic mesh with no comparison, questions regarding the optimal type of mesh and position of mesh placement in complex abdominal wall reconstruction cannot be fully answered. Conversely, the present study is strengthened by the relatively homogeneous patient population with a complex and contaminated abdominal wall defect and the use of a single biologic mesh. Our results should enable future comparisons and pooling of data, thereby elucidating the potential role of biologic meshes in complex abdominal wall repair. The results of this study suggest that repair of the most challenging abdominal wall defect can be done effectively with combination of a non-cross-linked biologic mesh and component separation technique without the need for mesh removal despite wound infections. No firm conclusions could be drawn regarding the durability of these repairs in terms of hernia recurrence, as follow-up was limited. However, the main focus of complex abdominal wall repair in contaminated fields is to safely perform this repair despite (severe) contamination due to either infected mesh, intestinal fistula or stoma reversal.
